# Approximate Depth Shape Reconstruction for RGB-D Images Captured from HMDs for Mixed Reality Applications

**DOI:** 10.3390/jimaging6030011

**Published:** 2020-03-05

**Authors:** Naoyuki Awano

**Affiliations:** Faculty of Business Administration, Osaka University of Economics, 2-2-8, Osumi, Higashiyodogawa-ku, Osaka 533-8533, Japan; awanonaoyuki@gmail.com

**Keywords:** depth image, head-mounted display, RGB-D, inpainting, mixed reality

## Abstract

Depth sensors are important in several fields to recognize real space. However, there are cases where most depth values in a depth image captured by a sensor are constrained because the depths of distal objects are not always captured. This often occurs when a low-cost depth sensor or structured-light depth sensor is used. This also occurs frequently in applications where depth sensors are used to replicate human vision, e.g., when using the sensors in head-mounted displays (HMDs). One ideal inpainting (repair or restoration) approach for depth images with large missing areas, such as partial foreground depths, is to inpaint only the foreground; however, conventional inpainting studies have attempted to inpaint entire images. Thus, under the assumption of an HMD-mounted depth sensor, we propose a method to inpaint partially and reconstruct an RGB-D depth image to preserve foreground shapes. The proposed method is comprised of a smoothing process for noise reduction, filling defects in the foreground area, and refining the filled depths. Experimental results demonstrate that the inpainted results produced using the proposed method preserve object shapes in the foreground area with accurate results of the inpainted area with respect to the real depth with the peak signal-to-noise ratio metric.

## 1. Introduction

Head-mounted displays (HMDs) have attracted increasing attention in many fields owing to their effectiveness in displaying virtual worlds. Representative types of HMDs fall into two categories, i.e., the see-through method to provide computer graphics (CG) in the real world and the non-see-through method, which covers the entire viewing field to provide virtual worlds [1,2,3]. Therefore, it is necessary to select a device that is appropriate for the target application.

HMDs are mainly used in applications in the fields of virtual reality (VR), augmented reality (AR), and mixed reality (MR). VR applications present only virtual worlds, giving users simulated experiences; AR applications overlay CGs onto real-world objects and augment information from the real world; and MR applications present an experience that can interact with both real and virtual worlds. However, HMDs face issues that must be addressed as HMDs become increasingly popular, such as their physical weight, size, display resolution, and interface. Many manufacturers have produced dedicated user interfaces for specific applications [4,5,6]. Furthermore, a camera with depth sensors can be mounted onto several HMDs to provide a more natural user interaction between the real and virtual worlds because depth sensors capture the distances to real objects as a depth image.

Depth sensors can facilitate real-space recognition more effectively than color images. For example, the hand regions in an image can be extracted easily by isolating distant depths, which can then be applied to hand recognition tasks. While this approach is effective for hand recognition, the associated image depths should not be removed in cases where the users directly interact with real objects in the virtual world, such as scanning objects, scanning the spaces surrounding users, and using devices in virtual worlds. Therefore, it is important to utilize all depths obtained from a depth sensor in MR applications effectively.

**Figure 1:**
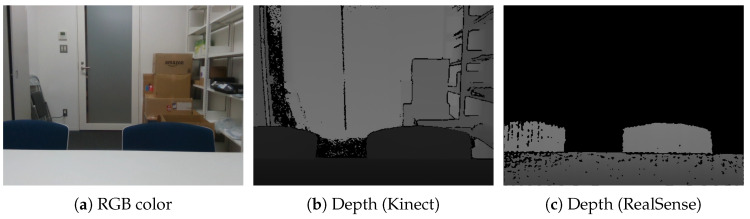
Resultant depth images obtained using different devices. The black pixels in each image define areas where the depth information could not be measured. (**a**) RGB color; (**b**) depth (Kinect); (**c**) depth (RealSense).

The representative methods to generate a depth image using depth sensors are structured-light (SL), time-of-flight (TOF), and the other stereo methods [7]. SL sensors obtain depth information through triangulation; TOF sensors obtain depth information by measuring the travel time of a light pulse; and the stereo methods estimate depth based on a stereo matching technique. There are cases when many pixels in a depth image captured by sensors do not possess depth information, such as those shown in Figure 1, because the depths to distal components in a given scene are not always captured. Although this often occurs with low-cost or SL sensors, it also occurs when a given scene contains many distal components, such as background elements. Because HMDs are required to be unobtrusive and lightweight, HMD-mounted sensors cannot avoid being small and low in cost. In addition, many distal components are included in applications that use the depth sensor of an HMD to replicate human vision. However, it is difficult to inpaint (repair or restore) large missing regions in depth images, just as the background cannot be made from the foreground.

In this paper, under the assumption of an HMD-mounted depth sensor, we propose a method to inpaint a depth image containing large missing regions. The proposed method resolves a scene that contains large missing regions when the pixels corresponding to the background do not possess depth information, e.g., an MR application to operate objects in both virtual and real space.

In the proposed method, we partially inpaint missing regions and reconstruct the shapes of only the foreground components. Conventional studies have primarily focused on inpainting an entire depth image to generate a complete image. Hence, a clear distinction must be made between the conventional approaches and the proposed inpainting approach in terms of whether they eventually create a complete image.

The proposed method first applies smoothing filters to reduce noise. Then, shapes in the foreground of the image are interpolated and reconstructed. Finally, the proposed method refines the interpolated depths to improve image accuracy.

## 2. Related Work

Until approximately one decade ago, depth sensors had primarily been used in reverse engineering. Such sensors, also known as 3D scanners or digitizers, have traditionally been very expensive, which has limited their practical applications. The recent development of the Kinect by Microsoft, which is an inexpensive depth sensor for the entertainment industry, has led to various fields adopting this sensor for numerous image recognition applications [8]. Depth sensors capture the distances from the sensor to real objects as a depth image. The Kinect can also capture RGB color images because it has a mounted RGB camera. The captured RGB color and depth images are generally referred to as RGB-D images.

As described in the previous section, SL and TOF are representative methods to obtain depth information. SL sensors obtain depth information via triangulation, and TOF sensors obtain depth by measuring the travel time of a light pulse. The depth information obtained by the SL method can theoretically demonstrate higher average accuracy at close distances than the TOF method, although the depths of the occluded regions are lacking [7]. The TOF method does not suffer as much from occlusion as the SL method; however, the TOF method does suffer from the effect of indirect bounces [9]. In addition, stereo matching-based sensors have been proposed [10]; however, their depth images also suffer problems related to occlusion.

Figure 1a,b illustrates RGB-D images captured using a Kinect v2 depth sensor (TOF). The intensity of the depth image in Figure 1b indicates the objects’ distances from the sensor, and black regions indicate that depth could not be measured. The black regions occur due to transparent, specular-reflected, and occluded objects, as well as components that are proximal or distal to the depth sensor, which are difficult to measure because depth sensors use infrared rays.

Depth sensors smaller than the Kinect have also been developed recently [11,12,13]. Figure 1c shows a depth image captured using the RealSense SR300 depth sensor (SL) developed by Intel. The RealSense’s depth image contains many more black pixels compared to the Kinect’s depth image because the RealSense cannot measure the background and distal objects. Therefore, an appropriate device must be used relative to an application’s requirements. In addition, it is also important to inpaint depth images properly according to the requirements of different applications.

In consideration of many factors, such as device selection, depth estimation methods, and environmental conditions, previous studies have proposed various inpainting approaches. For example, methods that use stereo images have been proposed as a disparity map inpainting method based on an approach that exploits the characteristic of stereo images [14,15]. In addition, methods have been proposed to align the edges of a depth image with those of a color image by exploiting the assumption that depth and color edges at corresponding locations are consistent [16,17,18] and, moreover, considering surface smoothness [19]. Furthermore, methods that adopt and extend the fast marching method, which is a numerical method for solving boundary value problems, have also been proposed [20,21]. Relative to the exemplar-based or patch-based approach, methods based on copying a similar source patch into a target patch of depth holes have also been proposed [22,23,24,25]. Various filtering-based methods have also been proposed previously, such as joint-bilateral filtering, which includes spatial and temporal information [26], an edge-preserving filter, which considers edges to refine straight lines [27], the adaptive hole-filling filter, which labels holes as areas of occluded or glossed objects [28], a dedicated bilateral-based filter, which considers visual distances in the L*a*b color space [29], and a median-based filter that applies improved bilateral and non-local means (NLM) for denoising [30]. In addition, many other methods have been proposed, such as the method based on 3D reconstruction with plane fitting and optimization [31], the linear anisotropic diffusion method, whose conductivity is designed based on a color image [32], and a method to inpaint depth images obtained using a Kinect underwater [33]. These methods are employed to fill and smooth holes and disocclusion areas, and they have demonstrated good results. Essentially, these methods share a common goal, i.e., inpainting an entire depth image to generate a complete image.

As described at Section 1, this study assumes the use of an HMD-mounted depth sensor. The mounted depth sensor’s accuracy is very low; thus, some approaches have attached a different depth sensors to HMDs for more interactions and improved accuracy [34,35]. However, there are some cases, such as that shown in Figure 1c, where the black area represents the background rather than holes, especially when using low-cost or SL sensors. Therefore, in this paper, we propose an ideal method that only inpaints the foreground and preserves the shapes of foreground objects.

## 3. Depth Image Inpainting Using an HMD-Mounted RGB-D Camera

### 3.1. Overview

The objective is to inpaint the depth image partially with numerous uncaptured depths, as shown in Figure 1c, especially in the case of using an HMD-mounted RGB-D camera. Note that the proposed method is not intended to generate a complete depth image.

**Figure 2:**
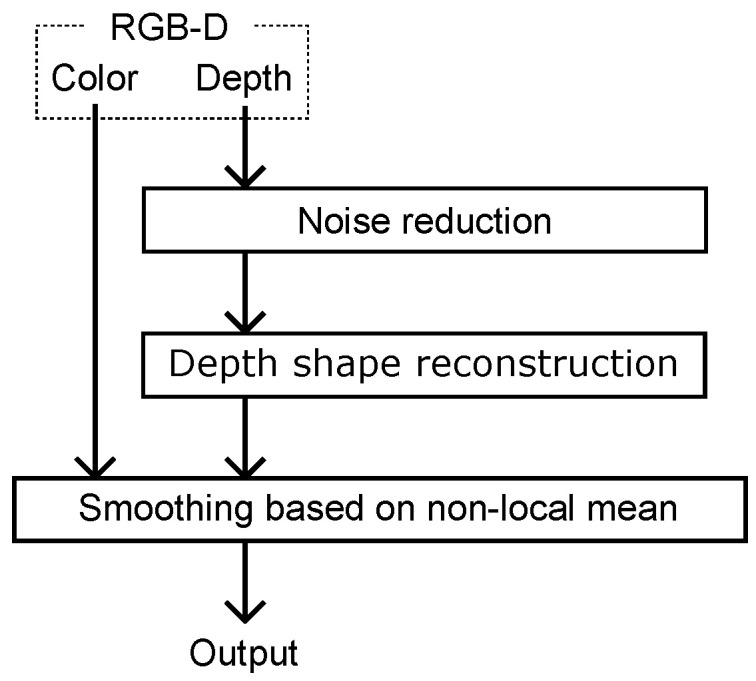
Outline of the proposed method.

Figure 2 shows the outline of the proposed method. First, we reduce noise in the depth image of the input RGB-D. Then, we distinguish the proximal area that should be inpainted and the other area, and we inpaint only the proximal area as depth shape reconstruction. Using the interpolated depth and color image, we apply an NLM-based smoothing filter to the interpolated proximal area.

In some MR applications, it is important to preserve only the shape of very close depth pixels rather than performing inpainting, such as requiring hand recognition or recognizing an object with hands. To address this issue in a simple manner, we propose a method to distinguish a very close area of the proximal area to apply the preceding method separately to the very close area and the other proximal area. If necessary, this approach is applied between the noise reduction and the interpolation.

### 3.2. Noise Reduction

First, it is necessary to reduce noise because raw depth images generally include spike noise. The median filter is as an effective method to reduce spike noise. In reference to previous studies [29,30], we employ the median filter as a pre-processing, and we apply it $N_{m}=3$ times to the entire depth image.

### 3.3. Depth Shape Reconstruction

**Figure 3:**
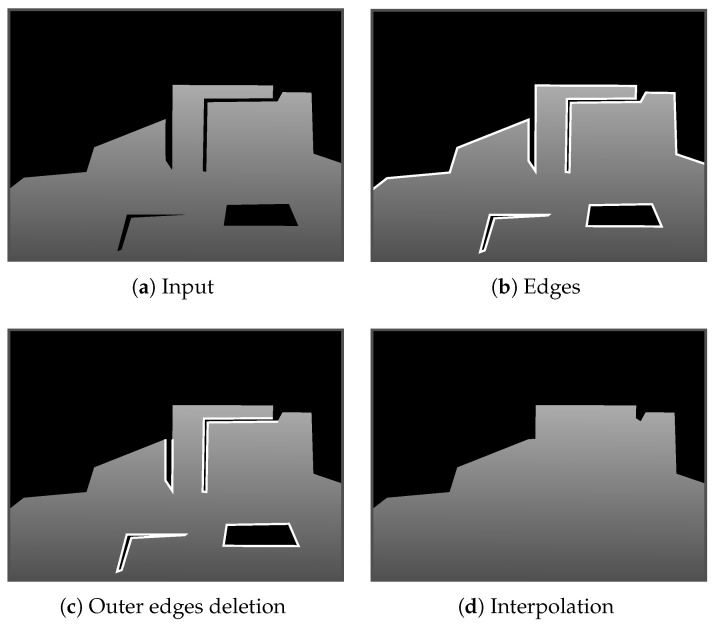
Overview of proposed inpainting approach. (**a**) Black areas indicate areas with no detected depth. (**b**) White lines mark extracted edges. (**c**) Outer edges are eliminated. (**d**) Interpolation using the edges in (**c**).

This approach distinguishes areas that should and should not be interpolated rather than distinguishing the foreground and background. Figure 3 illustrates our inpainting approach. First, we defined all pixels with depth. Then, we extracted the edge pixels $E=\{e_{i}\in R^{2}|1\leq i\leq N_{e}\}$ adjacent to black pixels, as shown in Figure 3b. Next, we only extracted edges enclosed by the depth pixels or constitute deep concave as $\hat{E}$ (Figure 3c). Finally, we applied Delaunay triangulation [36] to $\hat{E}$ and filled the holes with triangles, as demonstrated by [37]. Note that all the triangles were rendered to the depth image using the colors converted from the edge pixel depths. Then, the inside color of each triangle was interpolated linearly.

**Figure 4:**
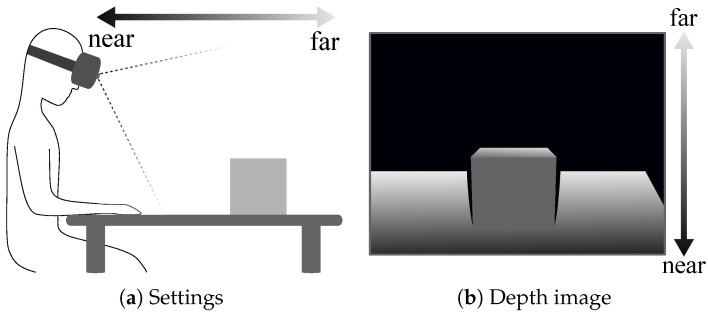
Composition of depths in depth images captured using a head-mounted display (HMD)- mounted depth sensor. (**a**) Settings; (**b**) depth image.

The core of this approach is the extraction of edges $\hat{E}$. This is the same as removing edges other than $\hat{E}$; thus, we extracted the edges other than $\hat{E}$ as outer edges and deleted them from *E*. Specifically, we utilized the depth attribute of the HMD-mounted depth images. As shown in Figure 4, proximal objects are toward the bottom of the image, whereas distal objects are toward the top. This illustrates the situation whereby most captured depths are concentrated at the bottom of the image, where distal objects are not captured. Therefore, the depth image was almost divided into two regions, i.e., a proximal region with depth and a distal region without depth. The shape of the proximal region should be inpainted because it is the foreground. In contrast, the shape of the region without depths should not be inpainted because the required depths do not exist. For example, holes in the proximal region and pixels with deep concavity should be inpainted while preserving the approximate shape because we assumed that they were in the foreground. Therefore, we extracted edge pixels that should not be inpainted and eliminated them from *E*. We then applied Delaunay triangulation.

**Figure 5:**
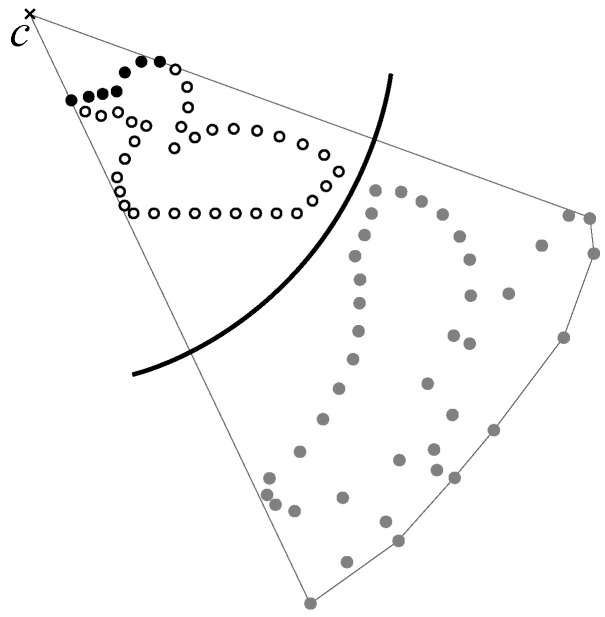
The hidden point removal (HPR) operator (black points indicate pixels visible from viewpoint *c*).

We used the hidden point removal (HPR) operator [38,39,40,41,42] and extended it so that it was applicable to our pixel extraction method. HPR was proposed as a rendering and shadowing method for three-dimensional point clouds, and it achieved a visibility check for point clouds with no information about the connectivity between points. Figure 5 shows HPR in 2D point clouds, which was accomplished as follows.

(a) Generate a circle with center *c*, which is also a viewpoint, and radius *r*.
(b) Place input points inside the circle.
(c) Flip the input points in the circle using Equation (1).
  (1) $$f\left(e,c\right)=e-c+2\left(r-∥e-c∥\right)\frac{e-c}{∥e-c∥},$$
(d) Construct the convex hull of the flipped points and *c*.
(e) Points on the convex hull are judged to be visible from cat the points before flipping.

Note that it was difficult to extract deep concave points with HPR; thus, we took advantage of this characteristics to extract approximate shapes without deep concavity.

**Figure 6:**
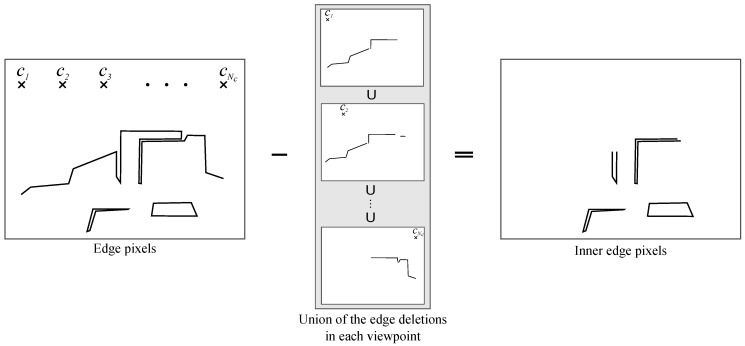
Inner-edge pixels after eliminating outer edges.

Figure 6 shows the outline for extracting inner-edge pixels that should be interpolated. First, the pixel locations of *E* were regarded as 2D points. Furthermore, although HPR set a single center point, we prepared multiple center points $C=\{c_{j}\in R^{2}|1\leq j\leq N_{c}\}$. The center points *C* were set along the upper side of the depth image, as shown in the left panel of Figure 6. Then, HPR was applied to *E* and $c_{1}$, and the edge pixels visible from $c_{1}$ were extracted. This visible edge pixel extraction process was conducted in parallel at the other center points. Finally, all extracted edge pixels were combined as outer edges and subtracted from the original edge pixels *E*, thereby extracting the inner-edge pixels (Figure 6, right panel).

The inner-edges $\hat{E}$ used for interpolation are formulated as follows.

(2) $$\hat{E}=E\backslash \left\{e_{k}^{\prime }\in E|F\left(f\left(e_{i},c_{j}\right),c_{j}\right)=1,e_{i}\in E,c_{j}\in C\right\},$$

(3) $$F\left(e,c\right)=\left\{\begin{array}{cc} 1 & \mathrm{if}\;e\;\mathrm{is}\;a\;\mathrm{vertex}\;\mathrm{of}\;\mathrm{the}\;\mathrm{convex}\;\mathrm{hull}\;\mathrm{of}\;H(c),\\ 0 & \mathrm{otherwise}. \end{array}\right.$$

(4) $$H\left(c\right)=\{h_{i}|f\left(e_{i},c\right),e_{i}\in E,1\leq i\leq N_{e}\}\cup c,$$

Finally, we applied Delaunay-based interpolation to $\hat{E}$ and rendered the triangles to the depth image.

### 3.4. Smoothing Based on Non-Local Means

It was necessary to sharpen or smooth the inside pixels of the triangles and accurately inpaint the object shapes because the holes were interpolated linearly in each triangle. This could be achieved using an edge-preserving smoothing filter, with the bilateral [43] and NLM [44] filters being representative examples. These filters were based on Gaussian weighting according to the differences in pixel values and distances. In the proposed method, we used the NLM filter because it achieved better results than the bilateral filter in most cases [45,46].

The general NLM approach targeted either single- or three-channel images. Although we primarily targeted a depth image (i.e., a single-channel image), we also included the RGB-D color image in the filter-weighting scheme to improve accuracy. Because the target depth images included large missing regions whose pixels did not possess depths, we proposed an NLM filter that applied only to the foreground region. Specifically, the proposed filter left the pixels of the missing regions intact and applied the weighted mean filter to the pixels with depths based on their similarity to adjacent pixels. Therefore, the edge-preserving smoothed depth g(x,y) is expressed as follows:

(5) $$g\left(x,y\right)=\left\{\begin{array}{cc} \frac{\sum_{m=-w}^{w}\sum_{n=-w}^{w}W(x,y,m,n)D(x+m,y+n)}{\sum_{m=-w}^{w}\sum_{n=-w}^{w}W(x,y,m,n)}\; & \mathrm{if}\;D(x,y)\;\mathrm{has}\;a\;\mathrm{depth},\\ 0 & \mathrm{otherwise}. \end{array}\right.$$

(6) $$\begin{array}{cc} W\left(x,y,m,n\right)=\sum_{s=-w^{\prime }}^{w^{\prime }}\sum_{t=-w^{\prime }}^{w^{\prime }} & [R^{\prime }\left(x,y,m,n,s,t\right)\\ \; & \times G^{\prime }\left(x,y,m,n,s,t\right)\\ \; & \times B^{\prime }\left(x,y,m,n,s,t\right)\\ \; & \times D^{\prime }\left(x,y,m,n,s,t\right)\\ \; & \times T(x,y,m,n,s,t)], \end{array}$$

(7) $$R^{\prime }\left(x,y,m,n,s,t\right)=exp(-\frac{{\left[R\left(x+s,y+t\right)-R\left(x+m+s,y+n+t\right)\right]}^{2}}{2\sigma^{2}}),$$

(8) $$G^{\prime }\left(x,y,m,n,s,t\right)=exp(-\frac{{\left[G\left(x+s,y+t\right)-G\left(x+m+s,y+n+t\right)\right]}^{2}}{2\sigma^{2}}),$$

(9) $$B^{\prime }\left(x,y,m,n,s,t\right)=exp(-\frac{{\left[B\left(x+s,y+t\right)-B\left(x+m+s,y+n+t\right)\right]}^{2}}{2\sigma^{2}}),$$

(10) $$D^{\prime }\left(x,y,m,n,s,t\right)=exp(-\frac{{\left[D\left(x+s,y+t\right)-D\left(x+m+s,y+n+t\right)\right]}^{2}}{2\sigma^{2}}),$$

(11) $$T\left(x,y,m,n,s,t\right)=\left\{\begin{array}{cc} 1 & \mathrm{if}\;D(x+m+s,y+n+t)\;\mathrm{has}\;a\;\mathrm{depth},\\ 0 & \mathrm{otherwise}, \end{array}\right.$$

where *w* is the size of the filter window, $w^{\prime }$ is the window size for similarity, σ is the standard deviation of the Gaussian distribution, and R(·), G(·), B(·), and D(·) are the R, G, B, and D pixel values, respectively. In this study, we set w=4, $w^{\prime }=1$, and σ=0.2 (when the *R*, *G*, *B*, and *D* values were normalized in the range [0,1]).

### 3.5. Definition of Closer Area

It is important to only preserve the shape of very close depth pixels in some MR applications using HMD, such as hand recognition and recognizing an object with hands. Pixels surrounding the contour of the very close depth area should not be interpolated linearly with the other area. Therefore, we distinguished the closer depth area and other depth areas. Then, we separately applied the interpolation, to each area. Specifically, we defined the closer area as hands and the continuous region connected to the hands.

**Figure 7:**
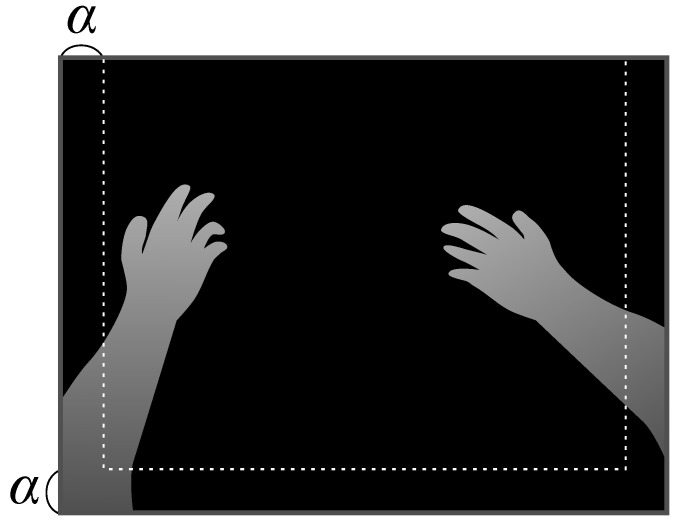
Defining width α to distinguish the closer area.

As shown in Figure 7, the hands were assumed to be at the front side of the image captured using an HMD-mounted camera. Here, we set the width α to the depth image in advance based on this assumption. First, pixel *p*, which had the nearest depth within $T_{d}$, was extracted in the area defined by α. Here, $T_{d}$ was the distance from the HMD-mounted camera to the threshold. Next, we applied the flood fill method [47] to *p* as the starting node. We then defined the filled area as the closer area. If both hands were present in the image, this procedure was repeated for the other hand after excluding the pixels of the already extracted closer area. The closer area may be separated due to noise or obstruction; thus, this procedure was repeated until *p* could not be extracted.

Then, we applied dedicated interpolation to the adjacent triangles connected to the extracted closer area. When one or two of the three vertices (i.e., pixels) of a triangle were in the closer area, the depths of these vertices were replaced with the other depth. For example, when only a vertex v1 of a triangle was in the closer area, its depth d1 was replaced with max(d2,d3), where d2 and d3 were the depths at the other vertices. Furthermore, when vertices v1 and v2 of a triangle were in the closer area, both d1 and d2 were replaced with d3. Finally, when all three vertices of a triangle were in the closer area, the triangle was eliminated because it existed on a finger or hand.

In this study, we set α to 5% of the image width. Furthermore, $T_{d}$ must be adjusted according to objective requirements because it depended on the positions of the HMD-mounted depth camera.

## 4. Results

We implemented the proposed method and conducted experiments to verify its capability and effectiveness. All experiments were performed using an Intel Core i7-8800K processor with 16 GB RAM and an NVIDIA GeForce GTX 1080. In addition, unless otherwise described, we used an HMD-mounted Intel RealSense SR300 RGB-D sensor with 640×480 pixel resolution.

### 4.1. Experimental Results

**Figure 8:**
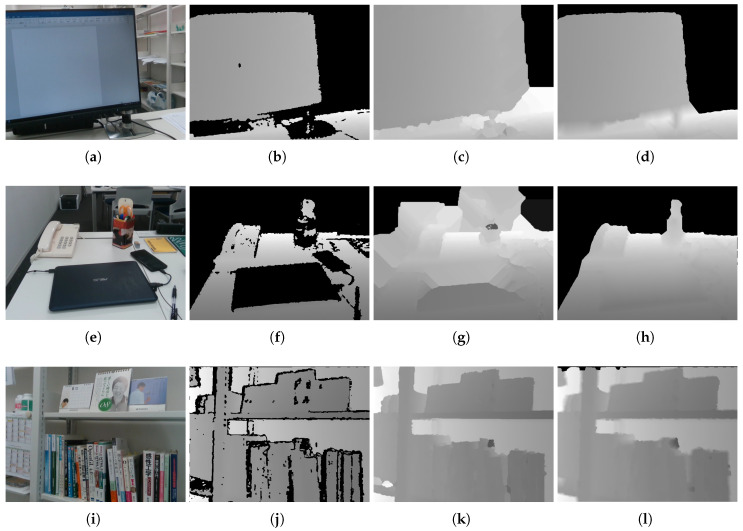
Inpainting results for three different scenes: (**a**,**e**,**i**) input color images; (**b**,**f**,**j**) raw depth images; (**c**,**g**,**k**) inpainting results obtained by the approach proposed by [30]; and (**d**,**h**,**l**) inpainting results obtained by the proposed method.

Figure 8 shows example results. As discussed in Section 1, the proposed method could not be compared with the other methods as to whether an image had been partially inpainted, because the conventional studies’ objective was not partial inpainting. However, to facilitate reasonable comparison, we compared an existing method [30] to highlight the difference in the results. We selected the method proposed by Bapat et al. because it partially inpainted the depth image depending on threshold; however, note that this partial inpainting was unintended by them. The top two rows in Figure 8 are scenes that included many uncaptured areas that were either too distal or contained darker objects. In these cases, the proposed method achieved good results relative to preserving foreground contour shapes, where our extended HPR operators preserved the shapes and obtained important results relative to our objective. The bottom row is a scene that contained relatively proximal objects such that the depth image included few uncaptured areas. In this case, most of the uncaptured areas were interpolated. A black portion of the area to the top of the image remained because the proposed method preserved the contours for the outer concavity. However, the proposed method still provided a good result in this case because many of the important areas were interpolated appropriately.

**Figure 9:**
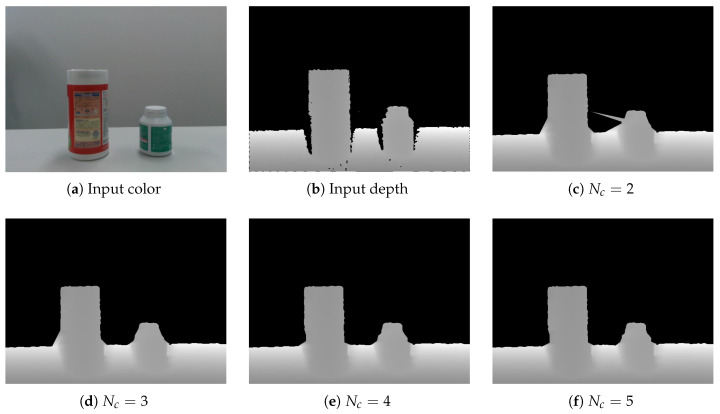
Results with the different $N_{c}$. (**a**) Input color; (**b**) input depth; (**c**) $N_{c}=2$; (**d**) $N_{c}=3$; (**e**) $N_{c}=4$; (**f**) $N_{c}=5$.

Figure 9 shows several results obtained with different values for parameter $N_{c}$, which was the number of the center points in our extended HPR. As can be seen, the shape was improved when $N_{c}$ was large. However, increasing $N_{c}$ to very large values did not improve the results. From this and the other preliminary results, we determined that $N_{c}=5$ was sufficient relative to performance improvement. Note that all subsequent results were obtained with $N_{c}=5$.

**Figure 10:**
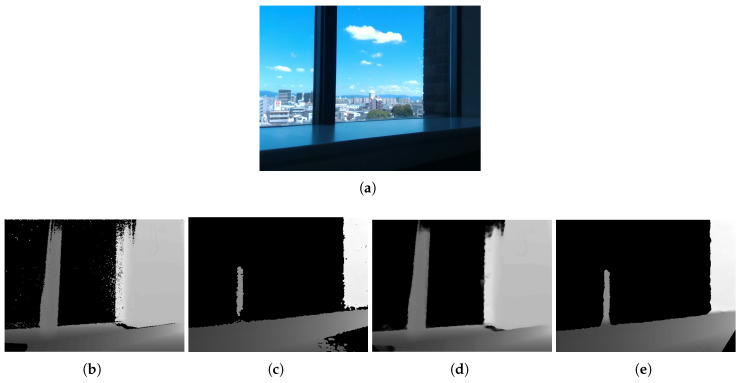
Inpainting results obtained with different sensors: (**a**) capture scene; (**b**) raw depth image from Kinect v2 (TOF); (**c**) raw depth image of RealSense SR300 (SL); and (**d**,**e**) inpainting results of (**b**,**c**).

Figure 10 shows the results for a case using different depth sensors. The Kinect depth image contained noise and incorrect depths due to the effect of indirect bounces. Therefore, the resultant image included some smoothless or incorrect inpainting regions. The RealSense depth image had only closer depths than the Kinect’s. Captured regions were relatively less, although the result showed preserving shapes in the region captured. Although there were differences due to sensor characteristics, the proposed method could obtain good results in terms of shape preservation.

**Figure 11:**
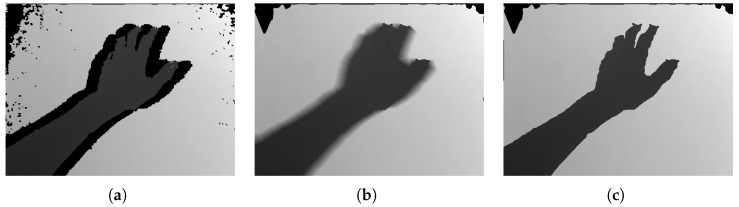
Closer depth area refinement results: (**a**) input depth image; (**b**) result without refinement; and (**c**) result with refinement.

Figure 11 shows the result of closer depth area refinement. As shown, the hand was blurred in the no refinement case (Figure 11b) because the uncaptured areas were primarily interpolated using a linear interpolation. In contrast, the hand shape was clearly preserved in the case with refinement (Figure 11c), which is important in some applications, such as hand recognition, which highlighted the effectiveness of this refinement. Note that this result depended on threshold $T_{d}$ (Section 3.5), which was the distance from the HMD-mounted camera positions. Here, we set $T_{d}=350$ (mm).

### 4.2. Objective Evaluation

**Figure 12:**
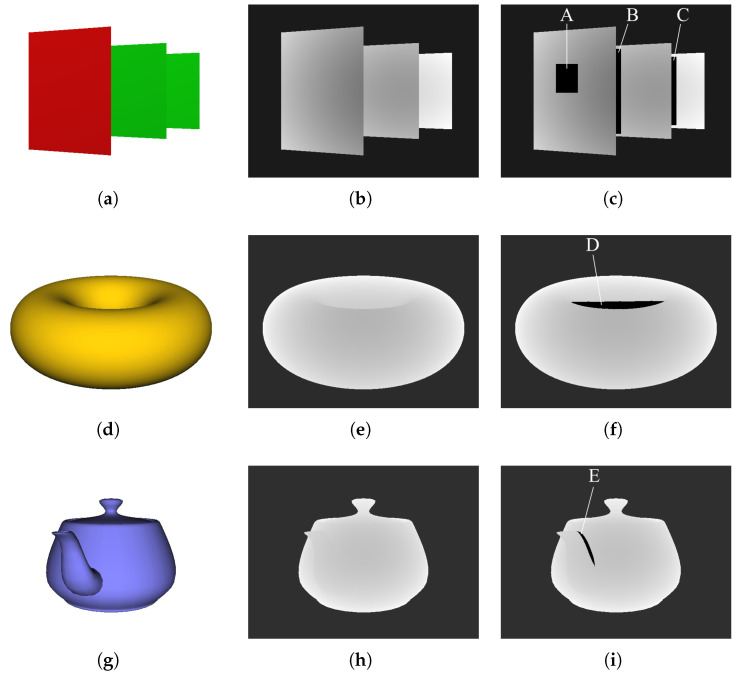
Three experimental images: (**a**–**c**) three planes, (**d**–**f**) torus, and (**g**–**i**) teapot. (**a**,**d**,**g**) Color images. Each image is a 3D CG object. (**b**,**e**,**h**) Depth maps. (**c**,**f**,**i**) Depth maps with eliminated areas.

A common method to evaluate image quality quantitatively is to calculate the peak signal-to-noise ratio (PSNR). For example, Bapat et al. [30] applied the PSNR to evaluate the accuracy of interpolated depths, where they eliminated some depths, inpainted the eliminated area, and then calculated the PSNR based on the difference before and after inpainting. However, this approach is often inappropriate because raw depth images generally include considerable noise that cannot be ground truthed; thus, the noise must be suppressed to achieve high accuracy. Therefore, we simulated some normal situations by placing them onto the depth maps of CG models rather than actual depth images. Here, we specifically created 3D CG objects and their depth maps for these normal situations, as shown in Figure 12. Furthermore, we eliminated a portion of each depth map to imitate actual situations. Then, we inpainted these eliminated areas using the proposed method and calculated the PSNR using the depth maps with and without smoothing.

Table 1 shows the PSNR values for the inpainted depth maps obtained using the proposed method. The accuracy of Areas (B) and (C) were particularly lower than that of the other areas. The reason for this reduced accuracy was likely due to the large depth differences in and around the eliminated areas, which were indicated by the large standard deviations (SD) for the depths in and around the eliminated area (Table 2). In contrast, the accuracy of Areas (A) and (E) was higher with relatively low SD values. Therefore, it appeared that accuracy depended on the depth differences of the surrounding inpainting areas.

However, Area (A) demonstrated the highest accuracy, even though Area (E) possessed the lowest SD. As shown in Figure 12, Area (A) was a planar defect, and Area (E) was a defect due to the curved surfaces between the components of the teapot. Therefore, Area (A) had higher accuracy due to the employed linear interpolation, which was suitable for the square shape. Further improvements to accuracy include extending the linear approach to an adaptive, nonlinear interpolation process.

Furthermore, it could be confirmed that our edge-preserving smoothing approach, which was based on NLM, improved the accuracy in all results. This smoothing approach placed the depths of the linear interpolated areas closer to the actual objects, and the results highlighted the importance and necessity of the proposed method.

We also measured processing times to evaluate the proposed method. Here, processing time was measured for a minute, with a mean time of 42 ms (computational cost rate: noise reduction 15%, depth shape reconstruction 68%, smoothing 14%, and closer area extraction 3%).

### 4.3. Application Example

**Figure 13:**
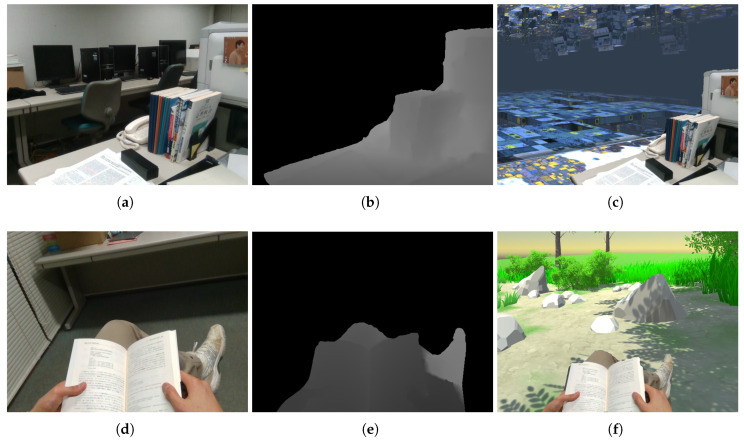
Two application examples: (**a**–**c**) a workspace and (**d**–**f**) reading in a small space. (**a**,**d**) Real-world images. (**b**,**e**) Depth images inpainted using the proposed method. (**c**,**f**) Composite results placed in virtual worlds.

The purpose of the proposed method was to inpaint depth images from an HMD-mounted RGB-D camera for use in MR applications. The specific procedure employed the HMD-mounted RGB-D camera to capture an RGB-D image, with the depth image subsequently inpainted using the proposed method. With the exception of areas with no depth constraints, the RGB-D image was mapped onto a virtual space and displayed using the HMD. Figure 13 illustrates business- and entertainment-related examples of this procedure, where the top row shows a workplace example first captured in real space and then mapped onto a virtual space. Although the workplace was a small office, it could readily be transformed into a spacious environment. The bottom row shows someone reading in a small space. Similar to the previous example, this small reading space could be transformed to a warm and spacious outdoor environment. Therefore, the proposed method could effectively create composites of the real and virtual worlds. Thus, we expected that the proposed method could be applied to various MR applications.

## 5. Conclusions

Various RGB-D sensors have been produced and adopted to achieve numerous objectives, such as user interfaces, object recognition, and object reconstruction. However, there are often cases where depth images contain defects that are too large, especially in low-cost or SL sensors. In this paper, under the assumption of an HMD-mounted depth sensor, we proposed a method to inpaint RGB-D depth images partially to preserve object shapes. The proposed method employed a denoising filter to reduce spike noise in the depth images. The proposed method clearly distinguished areas that should be inpainted using our extended HPR, which allowed us to avoid unnecessary inpainting processes. Furthermore, the proposed method improved the accuracy of inpainted depths using an edge-preserving smoothing filter. In addition, the proposed method applied a refinement process to closer areas, which is a common requirement in various MR applications.

We conducted experiments to confirm the effectiveness of the proposed method, and the results demonstrated effective inpainting that preserved the shapes of objects. We applied the proposed method to MR applications and highlighted its capabilities, as well as its potential use in other MR applications. While the proposed method was specialized to preserve the shapes of objects, there is room to improve the accuracy of inpainted depths. For example, an adaptive, nonlinear interpolation or the other approach can be employed to improve the accuracy of the proposed method since it primarily depends on the depth differences of surrounding inpainting areas. Furthermore, in the future, it will be necessary to evaluate the applicability and effectiveness of MR applications that implement the proposed method.

## Figures and Tables

**Table 1:** PSNR values (dB) of inpainted depth maps obtained using the proposed method. (A)–(E) indicate the eliminated areas in Figure 12.

Object	Without Smoothing	With Smoothing
Planes (A)	44.47	52.00
Planes (B)	17.78	19.61
Planes (C)	15.04	18.00
Torus (D)	24.80	33.98
Teapot (E)	42.05	50.45

**Table 2:** SD values for depths around the eliminated areas in Figure 12.

Object	Standard Deviation
Planes (A)	9.01
Planes (B)	24.86
Planes (C)	35.14
Torus (D)	11.16
Teapot (E)	4.22

## References

[1] Goradia I., Doshi J., Kurup L. (2014). A Review Paper on Oculus Rift & Project Morpheus. Int. J. Curr. Eng. Technol..

[2] Aruanno B., Garzotto F., Rodriguez M.C. HoloLens-based Mixed Reality Experiences for Subjects with Alzheimer’s Disease. Proceedings of the 12th Biannual Conference on Italian SIGCHI Chapter.

[3] Huber T., Wunderling T., Paschold M., Lang H., Kneist W., Hansen C. (2018). Highly immersive virtual reality laparoscopy simulation: Development and future aspects. Int. J. Comput. Assist. Radiol. Surg..

[4] Moro C., Stromberga Z., Raikos A., Stirling A. Combining Virtual (Oculus Rift & Gear VR) and Augmented Reality with Interactive Applications to Enhance Tertiary Medical and Biomedical Curricula. Proceedings of the SIGGRAPH ASIA 2016 Symposium on Education.

[5] Dodoo E.R., Hill B., Garcia A., Kohl A., MacAllister A., Schlueter J., Winer E. (2018). Evaluating Commodity Hardware and Software for Virtual Reality Assembly Training. Eng. Real. Virt. Real..

[6] Su Y., Chen D., Zheng C., Wang S., Chang L., Mei J., Zu Q., Hu B. (2018). Development of Virtual Reality-Based Rock Climbing System.

[7] Bouquet G., Thorstensen J., Bakke K.A.H., Risholm P. (2017). Design tool for TOF and SL based 3D cameras. Opt. Express.

[8] Lun R., Zhao W. (2015). A Survey of Applications and Human Motion Recognition with Microsoft Kinect. Int. J. Pattern Recognit. Artif. Intell..

[9] Sarbolandi H., Lefloch D., Kolb A. (2015). Kinect range sensing: Structured-light versus Time-of-Flight Kinect. Comput. Vis. Image Underst..

[10] Cabrera E.V., Ortiz L.E., da Silva B.M.F., Clua E.W.G., Goncalves L.M.G. (2018). A Versatile Method for Depth Data Error Estimation in RGB-D Sensors. Sensors.

[11] Chi W., Kono H., Tamura Y., Yamashita A., Asama H., Meng M.Q.H. A Human-friendly Robot Navigation Algorithm using the Risk-RRT approach. Proceedings of the IEEE International Conference on Real-Time Computing and Robotics.

[12] Carey N., Nagpal R., Werfel J. Fast, accurate, small-scale 3D scene capture using a low-cost depth sensor. Proceedings of the IEEE Winter Conference on Applications of Computer Vision.

[13] Fuersattel P., Plank C., Maier A., Riess C. (2017). Accurate laser scanner to camera calibration with application to range sensor evaluation. IPSJ Trans. Comput. Vis. Appl..

[14] Wang L., Jin H., Yang R., Gong M. Stereoscopic inpainting: Joint color and depth completion from stereo images. Proceedings of the 2008 IEEE Conference on Computer Vision and Pattern Recognition.

[15] Hervieu A., Papadakis N., Bugeau A., Gargallo P., Caselles V. Stereoscopic Image Inpainting: Distinct Depth Maps and Images Inpainting. Proceedings of the 2010 20th International Conference on Pattern Recognition.

[16] Chen W., Yue H., Wang J., Wu X. (2014). An improved edge detection algorithm for depth map inpainting. Opt. Lasers Eng..

[17] Zuo Y., Wu Q., Zhang J., An P. (2018). Explicit Edge Inconsistency Evaluation Model for Color-Guided Depth Map Enhancement. IEEE Trans. Circuits Syst. Video Technol..

[18] Zhang H.T., Yu J., Wang Z.F. (2018). Probability contour guided depth map inpainting and superresolution using non-local total generalized variation. Multimed. Tools Appl..

[19] Miao D., Fu J., Lu Y., Li S., Chen C.W. Texture-assisted Kinect depth inpainting. Proceedings of the 2012 IEEE International Symposium on Circuits and Systems.

[20] Liu J., Gong X., Liu J. Guided inpainting and filtering for Kinect depth maps. Proceedings of the 21st International Conference on Pattern Recognition (ICPR2012).

[21] Gong X., Liu J., Zhou W., Liu J. (2013). Guided Depth Enhancement via a Fast Marching Method. Image Vis. Comput..

[22] Gautier J., Le Meur O., Guillemot C. Depth-based image completion for view synthesis. Proceedings of the 2011 3DTV Conference: The True Vision—Capture, Transmission and Display of 3D Video (3DTV-CON).

[23] Doria D., Radke R.J. Filling large holes in LiDAR data by inpainting depth gradients. Proceedings of the 2012 IEEE Computer Society Conference on Computer Vision and Pattern Recognition Workshops.

[24] Reel S., Cheung G., Wong P., Dooley L.S. Joint texture-depth pixel inpainting of disocclusion holes in virtual view synthesis. Proceedings of the 2013 Asia-Pacific Signal and Information Processing Association Annual Summit and Conference.

[25] Ciotta M., Androutsos D. Depth guided image completion for structure and texture synthesis. Proceedings of the 2016 IEEE International Conference on Acoustics, Speech and Signal Processing (ICASSP).

[26] Massimo Camplani L.S. Efficient spatio-temporal hole filling strategy for Kinect depth maps. Proceedings of the Three-Dimensional Image Processing (3DIP) and Applications II.

[27] Schmeing M., Jiang X., Jiang X., Bellon O.R.P., Goldgof D., Oishi T. (2013). Color Segmentation Based Depth Image Filtering. Advances in Depth Image Analysis and Applications. WDIA 2012. Lecture Notes in Computer Science.

[28] Vijayanagar K.R., Loghman M., Kim J. (2014). Real-Time Refinement of Kinect Depth Maps using Multi-Resolution Anisotropic Diffusion. Mob. Netw. Appl..

[29] Ishii H., Meguro M. (2015). Hole Filter of Depth Data Using the Color Information.

[30] Bapat A., Ravi A., Raman S. An iterative, non-local approach for restoring depth maps in RGB-D images. Proceedings of the Twenty-First National Conference on Communications (NCC).

[31] Barron J.T., Malik J. Intrinsic Scene Properties from a Single RGB-D Image. Proceedings of the 2013 IEEE Conference on Computer Vision and Pattern Recognition.

[32] Liu J., Gong X., Huet B., Ngo C.W., Tang J., Zhou Z.H., Hauptmann A.G., Yan S. (2013). Guided Depth Enhancement via Anisotropic Diffusion. Advances in Multimedia Information Processing—PCM 2013.

[33] Lu H., Zhang Y., Li Y., Zhou Q., Tadoh R., Uemura T., Kim H., Serikawa S. (2017). Depth Map Reconstruction for Underwater Kinect Camera Using Inpainting and Local Image Mode Filtering. IEEE Access.

[34] Garon M., Boulet P.O., Doiron J.P., Beaulieu L., Lalonde J.F. Real-time High Resolution 3D Data on the HoloLens. Proceedings of the International Symposium on Mixed and Augmented Reality (ISMAR).

[35] Intel Software HELIOS-Enhanced Vision to Empower the Visually Impaired with Intel RealSense Technology. https://software.intel.com/en-us/videos/helios-enhanced-vision-to-empower-the-visually-impaired-with-intel-realsense-technology.

[36] Ruppert J. (1995). A Delaunay Refinement Algorithm for Quality 2-Dimensional Mesh Generation. J. Alg..

[37] Kurata S., Ishiyama Y., Mori H., Toyama F., Shoji K. (2013). Colorization of Freehand Line Drawings Using Reference Images. J. Inst. Image Inf. Telev. Eng..

[38] Katz S., Tal A., Basri R. (2007). Direct Visibility of Point Sets. ACM Trans. Graph..

[39] Mehra R., Tripathi P., Sheffer A., Mitra N.J. (2010). Visibility of Noisy Point Cloud Data. Comput. Graph..

[40] Chen Y.L., Chen B.Y., Lai S.H., Nishita T. (2010). Binary Orientation Trees for Volume and Surface Reconstruction from Unoriented Point Clouds. Comput. Graph. Forum.

[41] Katz S., Tal A. On the Visibility of Point Clouds. Proceedings of the IEEE International Conference on Computer Vision (ICCV).

[42] Katz S., Tal A. (2017). On visibility and empty-region graphs. Comput. Graph..

[43] Tomasi C., Manduchi R. Bilateral Filtering for Gray and Color Images. Proceedings of the Sixth International Conference on Computer Vision (ICCV).

[44] Buades A., Coll B., Morel J.M. A Non-Local Algorithm for Image Denoising. Proceedings of the IEEE Computer Society Conference on Computer Vision and Pattern Recognition (CVPR).

[45] Anh D.N. (2014). Iterative Bilateral Filter and Non-Local Mean. Int. J. Comput. Appl..

[46] Mould D. (2013). Image and Video Abstraction Using Cumulative Range Geodesic Filtering. Comput. Graph..

[47] Torbert S. (2016). Applied Computer Science.

